# Attentional responses to the color exposure sequence of landscape paintings: evidence from fNIRS and eye-tracking

**DOI:** 10.3389/fnhum.2025.1719224

**Published:** 2025-12-10

**Authors:** Hyoyeong Jeong, Seongdae Kim

**Affiliations:** 1 Department of Human Artificial Intelligence, Sangmyung University, Seoul, Republic of Korea; 2 Division of Biohealth Convergence, Hongik University, Seoul, Republic of Korea; 3 Department of Public Health, Korea University Graduate School, Seoul, Republic of Korea

**Keywords:** color, art appreciation, concentration, functional near-infrared spectroscopy, eye tracking, landscape paintings

## Abstract

**Introduction:**

Color is a critical determinant of esthetic experience, shaping both immersion and cognitive responses. However, the extent to which the sequence of color exposure modulates the interplay between neural activation and ocular behavior remains insufficiently understood.

**Methods:**

Ten chromatic landscape paintings were collected and converted into achromatic versions, yielding a total of 20 visual stimuli. Participants were assigned to two conditions: Condition A (achromatic to chromatic) and Condition B (chromatic to achromatic). Visual attention and prefrontal activation were assessed using eye-tracking techniques and functional near-infrared spectroscopy.

**Results:**

Analyses revealed significantly greater neural activation in Condition A within the left dorsolateral prefrontal cortex (*p*-raw = 0.006), left orbitofrontal cortex (*p*-raw = 0.043), and right orbitofrontal cortex (*p*-raw = 0.043). Eye-tracking metrics indicated longer Total duration of fixation (*p*-raw = 0.015), Maximum duration of fixation (*p*-raw = 0.036), and Total duration of whole fixation (*p*-raw = 0.025) under Condition A. Subjective evaluations also showed significant differences, with higher ratings for Match (*p*-raw = 0.005) and Feel (*p*-raw = 0.029).

**Discussion:**

These findings demonstrate that color stimuli exert a decisive influence not only on subjective immersion but also on objective neurocognitive processes. The study extends the scientific foundation of empirical esthetics and offers novel implications for the design of museum and exhibition environments.

## 1 Introduction

Color is one of the core factors that evoke immersion, esthetic preference, and cognitive responses during the appreciation of artworks (Albers et al., 2020). It can be broadly categorized into achromatic tones, which are expressed solely through brightness without hue or saturation, and chromatic tones, which incorporate hue, saturation, and brightness (Emi̇rteki̇n, 2018). Previous research has demonstrated that when approximately 400 chromatic and achromatic images were randomly presented in two separate sessions, chromatic images elicited significantly stronger emotional responses, amplifying both positive and negative affective states (Lin et al., 2024). Furthermore, color enriches esthetic experience by increasing the perceived number of compositional elements and, consequently, the visual complexity of an image. Importantly, an optimal level of complexity has been reported to enhance esthetic pleasure through heightened visual stimulation (Massaro et al., 2012). Thus, color serves as a critical expressive medium that bridges emotional and psychological domains with perceptual and cognitive processes (Yao et al., 2022; Hu et al., 2024).

Building on these findings, studies indicate that the sequential presentation of chromatic and achromatic stimuli shapes subsequent visual perception and color adaptation. Sequential exposure enables the brain to use the chromatic characteristics of preceding stimuli as a reference frame when processing new color information. Accordingly, comparing exposure orders provides insight into how the temporal dynamics of visual input modulate cognitive processing and neural activation beyond simple color-contrast effects (Zohdi et al., 2021). Moreover, even brief color exposure can rapidly modulate attentional and emotional responses, inducing changes in oxygenation within prefrontal and limbic regions (Scholkmann et al., 2017). Salient visual features such as color—acting in concert with luminance and contrast—facilitate stimulus-driven attentional engagement while concurrently evoking affective responses and higher-order processing (Bekhtereva and Müller, 2017).

In contemporary psychology, vision is considered the dominant sensory modality for acquiring information from the environment. It is estimated that over 80% of external information perceived by humans is transmitted visually, and within the first 20 s of processing, color accounts for nearly 80% of the perceptual attributes identified among visual features such as shape and texture. This underscores the central role of color in rapid recognition and higher-order visual cognition (Yao et al., 2022; Hu et al., 2024). Correspondingly, recent studies have increasingly utilized eye-tracking techniques to investigate selective attention, esthetic experience, and sensory formation in the context of art perception (Massaro et al., 2012; Hiramatsu et al., 2023). In the appreciation of artworks, eye-movement direction serves as a key indicator of visual attention, while the temporal and spatial sequence of fixations reflects which regions of a stimulus were observed and for how long. Consequently, the first Area of Interest (AOI) encountered is often interpreted as the region that captures the highest level of visual salience and attentional engagement (Punde et al., 2017).

Eye movements are closely coupled with cognitive processes, offering critical insight into the neural correlates of esthetic experience (Arioli et al., 2025). This coupling reflects the role of the cerebral cortex in recognizing and extracting visual features that, in turn, underpin higher-order reasoning. James described visual attention as a cognitive process shaped by expectations and interests concerning “what,” which entails the selective allocation of neural resources to specific features of a stimulus (Nissen, 2020). Despite this theoretical integration, most prior studies on art appreciation have primarily focused on statistical analyses of external factors such as illumination, brightness, or color (Feltrin et al., 2020; Lin et al., 2024). Given that esthetic judgment and perception are inherently subjective, a central challenge remains in determining how these dimensions can be systematically addressed through empirical approaches (Quian Quiroga and Pedreira, 2011).

In recent years, growing efforts have examined brain–behavior relationships in esthetic experience using mathematical and computational approaches alongside noninvasive neuroimaging such as electroencephalography, functional magnetic resonance imaging, and functional near-infrared spectroscopy (fNIRS) (Liu and Hong, 2015; Nakauchi et al., 2022). Among these, fNIRS has emerged as a practical optical method for probing prefrontal neural activity during cognitive and decision-making tasks and has been applied across diverse populations and contexts (Obrig and Villringer, 2003; Kaimal et al., 2017; Nissen, 2020). Notably, fNIRS has proven effective for delineating frontal activation patterns during art-based tasks and art therapy, with robust changes reported in the ventrolateral prefrontal cortex (VLPFC), orbitofrontal cortex (OFC), and dorsolateral prefrontal cortex (DLPFC) (Han et al., 2024). These findings converge on the central role of the prefrontal cortex in art perception and evaluative processes (Kaimal et al., 2017).

Furthermore, research has increasingly combined eye-tracking with fNIRS to examine the relationship between visual attention and neural activation. For example, Pinheiro et al. (2024) measured eye movement patterns and prefrontal oxygenation changes among children engaged in robot-based learning activities, demonstrating that such a multimodal approach enables a more precise characterization of attentional engagement and cognitive processing than single-modality methods alone. In esthetic and art-based contexts, fNIRS has consistently revealed robust activation in the VLPFC, OFC, and DLPFC during perceptual evaluation and expressive processes (Han et al., 2024), supporting the central role of the prefrontal cortex in meaning construction, appraisal, and affective response to artworks (Kaimal et al., 2017). Han et al. (2024) further reported that the DLPFC is engaged during high-level cognitive activities involving imagery-based representations and self-referential thinking, whereas the OFC is implicated not only in memory and semantic processing but also in the perceptual evaluation of visual stimuli and the attribution of subjective value (Han et al., 2024). Together, these findings indicate that prefrontal activity reflects the interaction between perceptual input and higher-order cognitive evaluation, making fNIRS-eye-tracking integration a suitable approach for studying esthetic attention (Nissen, 2020).

Accordingly, the present study aimed to investigate the sequential effects of color presentation order on attentional engagement and interest-based artwork selection during the viewing of landscape paintings. To this end, participants completed a task in which the chromatic and achromatic versions of the same artworks were presented in different sequences. By employing fNIRS in conjunction with eye-tracking, this study examined how temporal ordering of color stimuli influences cognitive processes such as attention, memory, and decision-making through the integrated assessment of prefrontal oxygenation responses and visual attention patterns. This approach extends the understanding of esthetic experience beyond subjective reports or descriptive evaluations by incorporating objective neurophysiological indicators. Moreover, by quantitatively analyzing viewers’ visual focus and cognitive responses, the findings contribute practical implications for museum and gallery settings, offering insights into how exhibition design may be informed by the cognitive dynamics of art appreciation (Nissen, 2020).

## 2 Methods

### 2.1 Participants

The experiment was conducted with 31 undergraduate students (10 males and 21 females) aged between 21 and 29 years. All participants received detailed information about the study and provided written informed consent prior to participation. The study was performed in accordance with the Declaration of Helsinki and was approved by the Institutional Review Board of Hongik University (IRB No. 7002340-202409-HR-023).

### 2.2 Experimental design

A total of 10 chromatic landscape paintings were selected as visual stimuli. To create corresponding achromatic versions, each chromatic image was converted into grayscale using the OpenCV function cv2.cvtColor. To minimize differences in luminance and contrast that could result from this conversion process, additional preprocessing was conducted using cv2.normalize and cv2.equalizeHist. These procedures standardized pixel intensity values within the 0 to 255 range and equalized brightness and contrast distributions. Through this preprocessing, average luminance and contrast were controlled across all stimuli, ensuring that any observed effects could be attributed to the presence of color rather than to unintended visual artifacts. As a result, 20 stimuli were produced, consisting of 10 chromatic images and 10 achromatic images, forming 10 matched stimulus sets.

The experiment was conducted using a within-subjects design. All participants viewed all 10 stimulus sets, with each set containing a chromatic image and its achromatic counterpart. The presentation order of the two images within each set was randomized. The two possible presentation orders were defined as Condition A: achromatic to chromatic and Condition B: chromatic to achromatic. The sequence of stimulus sets was also randomized across participants, and Latin square counterbalancing procedures ensured that exposure frequencies to each order were evenly distributed across the participant group. This approach minimized the influence of inter-individual variability and reduced potential order-related bias.

**Figure 1:**
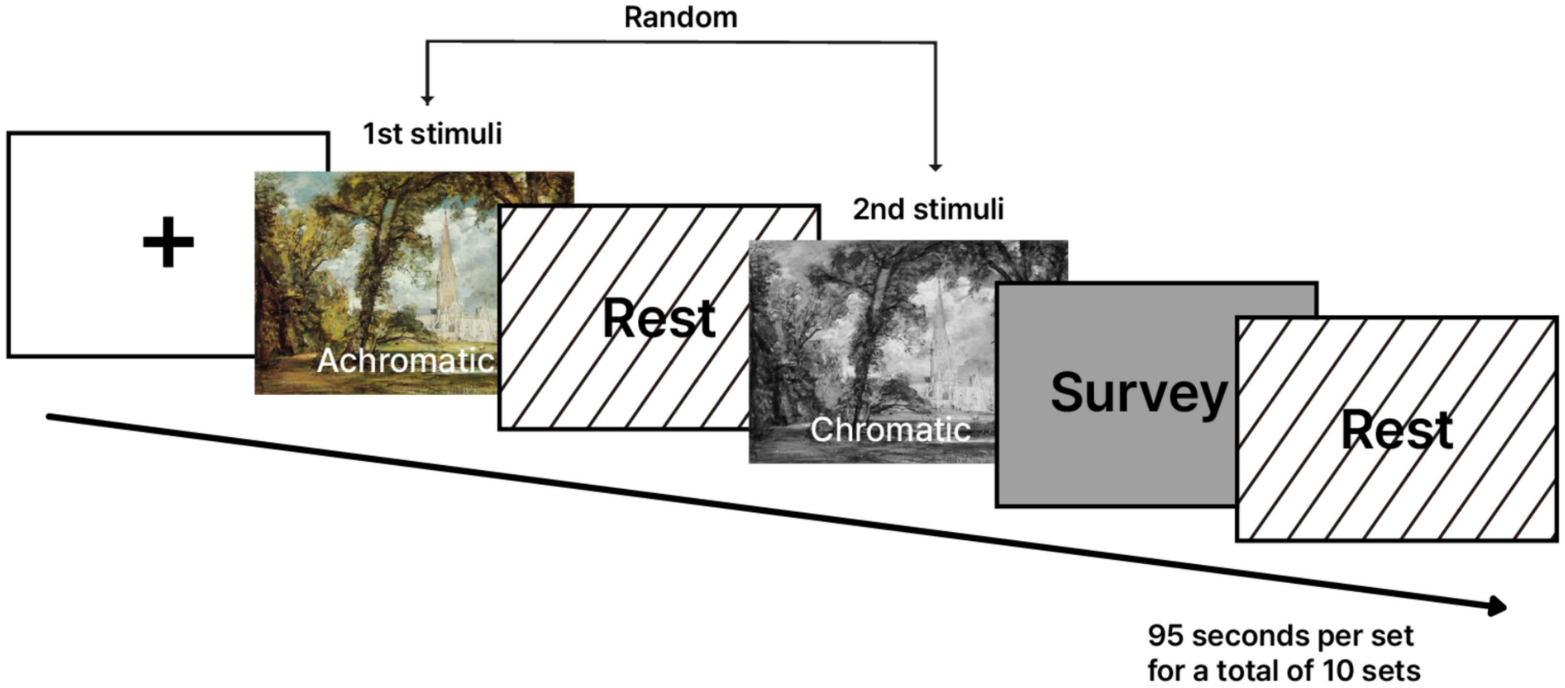
Experimental design. The first and second stimuli were identical landscape paintings, differing only in color (chromatic and achromatic). The presentation order of the two stimuli was randomized, and participants were not informed of the sequence in advance. Adapted with permission from Salisbury Cathedral from Lower Marsh Close by John Constable, licensed under CC0. Source: National Gallery of Art (Washington, DC) via Google Arts & Culture.

Prior to the experimental session, calibration was performed for both the eye-tracking system and the fNIRS. Participants were instructed to fixate on a centrally displayed point to stabilize gaze before stimulus presentation. Each stimulus sequence proceeded as follows. The first image in the set was presented for 15 s. The second image, presented in the opposite color condition, was then shown for 15 s. A rest period of 25 s followed to allow the hemodynamic response to return toward baseline. After the second image, participants completed a 15-second self-report assessment evaluating immersion and preference related to the visual experience. An additional 25-second rest period followed before the next set began. Each set required a total of 95 s to complete, and the full experimental session lasted approximately nine hundred fifty seconds, or about 15 min. Eye-tracking data were collected to quantify visual attention distribution, and fNIRS data were collected simultaneously to measure changes in prefrontal blood oxygenation associated with neural activity (Figure 1).

**Figure 2:**
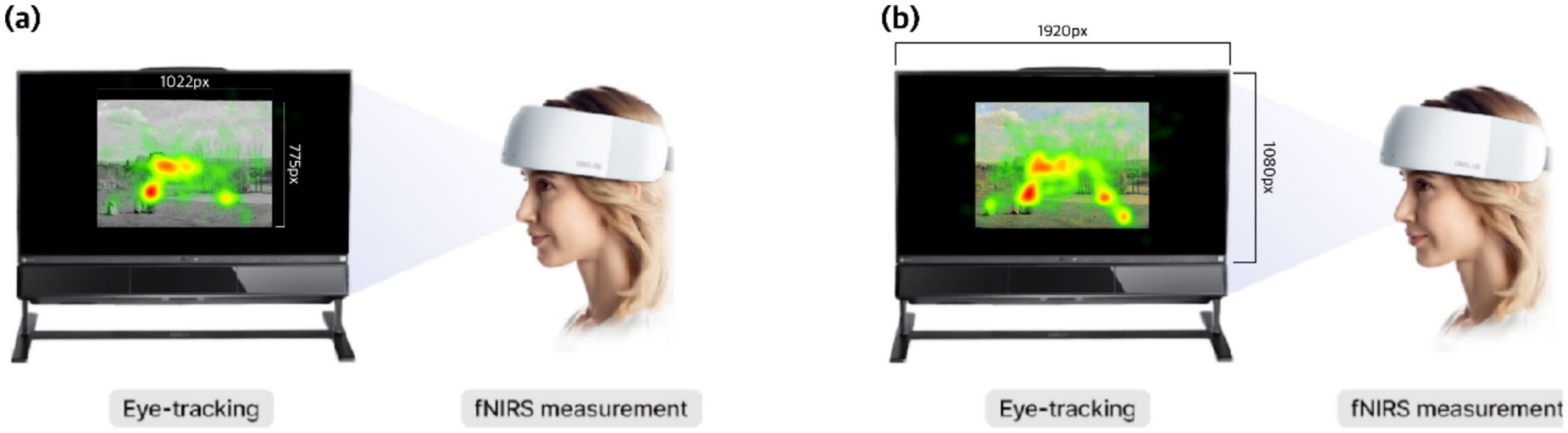
Configuration of eye-tracking AOI and overall fNIRS–eye-tracking measurement environment. This figure schematically illustrates our experimental design, in which fNIRS and eye-tracking were measured simultaneously. The left image **(a)** represents the achromatic stimulus, which was presented at a resolution of 1,022 × 775 pixels, and the entire image area was defined as a single AOI for the eye-tracking analysis. The right image **(b)** was displayed across the full monitor dimensions within the experimental setup to ensure consistent AOI calibration, accurate gaze mapping, and comparability of measurements across participants. This presentation sequence, in which the achromatic stimulus preceded the chromatic version, corresponds to Condition A in the experimental design. Adapted with permission from the OBELAB fNIRS device reference image by OBELAB Inc., licensed for use with attribution. Source: OBELAB homepage (www.obelab.com).

At the equipment level, visual attention and prefrontal hemodynamic responses were recorded concurrently to capture both behavioral and neural indicators of esthetic processing. This figure provides an overview of the experimental setup in which eye-tracking and fNIRS measurements were conducted simultaneously. The eye-tracking system captured gaze data directed toward the central AOI on the stimulus display, illustrating how visual attention was distributed during image viewing. At the same time, participants wore the NIRSIT fNIRS device, allowing for the measurement of hemodynamic responses in the prefrontal cortex while observing the same visual stimuli. This multi-modal approach enabled concurrent assessment of spatial patterns of visual attention, represented through fixation mapping, and neural activity associated with cognitive and affective processing, represented through fNIRS activation. Together, the setup demonstrates the capacity to examine correlations between attentional allocation and prefrontal cortical engagement during esthetic perception (Figure 2).

### 2.3 Rationale for the selection of artworks

Landscape paintings, compared with other types of artworks, depict a broader range of visual elements and therefore depend more extensively on color encompassing both chromatic and achromatic dimensions to convey complex information. Previous studies have highlighted the critical role of color in this process. Snowden demonstrated that color provides an effective cue for spatial localization and target detection, whereas Laarni reported that under high information-processing demands, color stimuli enhance both the speed and accuracy of visual responses. Furthermore, Wright proposed that color functions as a physical cue in the perception of depth and distance, and subsequent findings have provided empirical support for this view (Polzella et al., 2005).

### 2.4 Data acquisition

#### 2.4.1 fNIRS data

fNIRS data were collected using the NIRSIT device manufactured by OBELAB Inc. in Seoul, Republic of Korea. Functional near-infrared spectroscopy is a noninvasive neuroimaging method that measures changes in the oxygenation state of hemoglobin in the cerebral cortex by emitting light within the near-infrared range of approximately 650 to 1,000 nm and detecting the proportion of emitted light that is absorbed or reflected. The relative concentration changes in oxygenated hemoglobin (HbO) and deoxygenated hemoglobin (HbR) can be estimated using the modified Beer–Lambert law, enabling the assessment of hemodynamic responses associated with neural activity. When a brain region becomes more active, both total hemoglobin (HbT) and HbO typically increase, while HbR decreases (Wilcox and Biondi, 2015; Kamran and Hong, 2013; Kamran et al., 2016). This is possible due to the near-infrared optical window in which light absorption is minimized, allowing NIR light to penetrate biological tissue. Although HbR is also physiologically relevant, its signal tends to exhibit a lower signal-to-noise ratio than HbO, and its change during cognitive activation is often more gradual. Consequently, HbO has been widely regarded as the more sensitive indicator of task-induced cortical activation (Tak and Ye, 2014). While fNIRS does not directly quantify attentional intensity, prior research has shown that variations in HbO can be used to distinguish levels of cognitive load and engagement (Kang et al., 2021).

**Figure 3:**
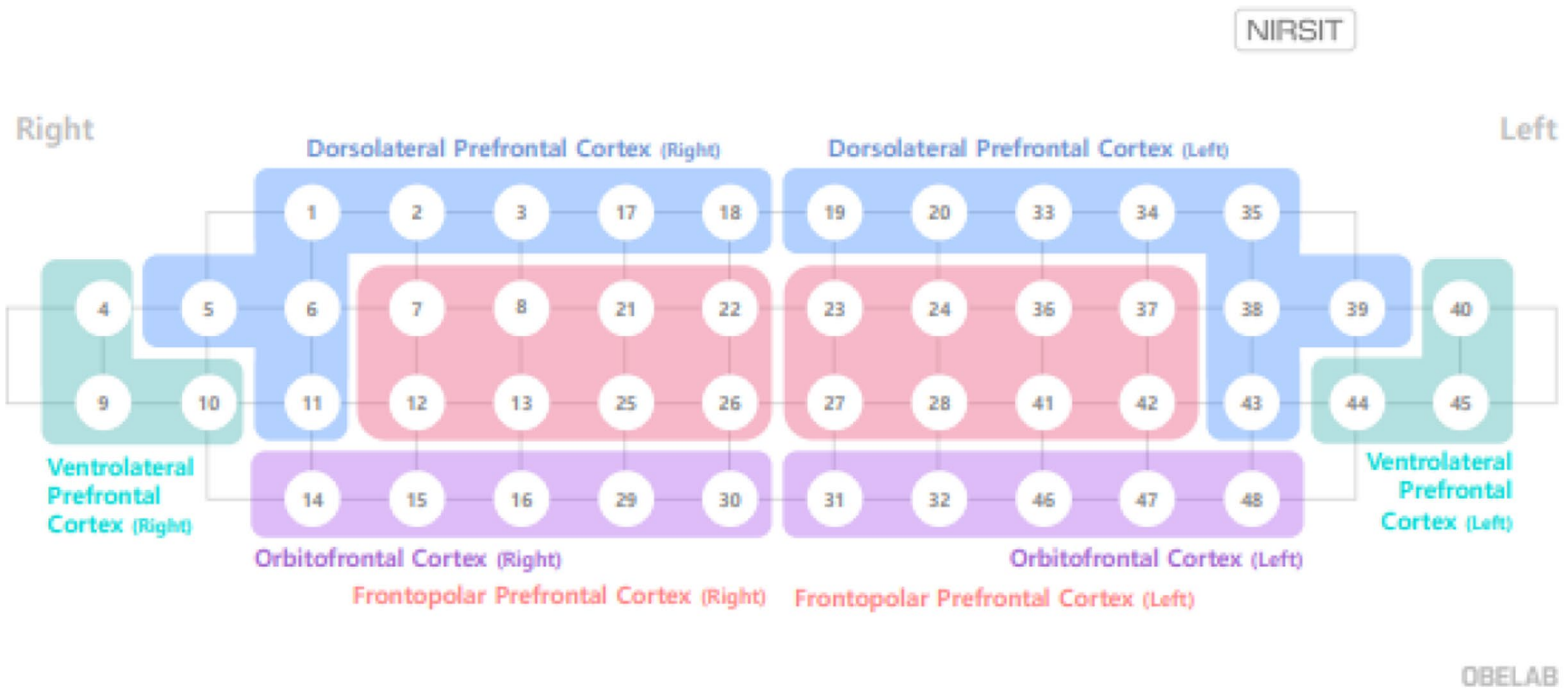
Configuration and measurement positions of the 48-channel NIRSIT fNIRS system. Right DLPFC (Right dorsolateral prefrontal cortex): 1, 2, 3, 5, 6, 11, 17, and 18 channels; Left DLPFC (Left dorsolateral prefrontal cortex): 19, 20, 33, 34, 35, 38, 39, and 43 channels; Right FPC (Right frontopolar cortex): 7, 8, 12, 13, 21, 22, 25, and 26 channels; Left FPC (Left frontopolar cortex): 23, 24, 27, 28, 36, 37, 41, and 42 channels; Right VLPFC (Right ventrolateral prefrontal cortex): 4, 9, and 10 channels; Left VLPFC (Left ventrolateral prefrontal cortex): 40, 44, 45.

Compared to fNIRS, functional magnetic resonance imaging provides high spatial resolution and detailed anatomical visualization but is limited by slow acquisition speed and reduced ecological validity. Electroencephalography offers high temporal resolution but suffers from low spatial resolution and sensitivity to artifacts. The NIRSIT system used in this study offers advantages in usability and portability, requiring neither conductive gels nor extensive sensor wiring. However, prolonged wear may introduce discomfort due to weight and head pressure (Ji et al., 2022). The device includes 32 light detectors arranged to produce 48 measurement channels at an inter-optode distance of 3.0 cm. As illustrated in Figure 3, these 48 channels were grouped into eight predefined cortical regions.

Preprocessing was conducted using the NIRSIT Analysis Tool provided by OBELAB Inc. Channels with invalid optical intensity values were automatically excluded. Motion artifacts were corrected using the Time-Domain Detrending Regression method, which detects abrupt signal fluctuations based on temporal gradients and removes them through robust regression. Signal quality evaluation followed three criteria. First, channels with optical intensity values below 30 arbitrary units were removed to avoid low-intensity signal bias. Second, channels with a coefficient of variation greater than 15 percent across the recording period were excluded due to signal instability. Third, channels in which identical optical intensity values appeared continuously for more than 5 percent of the recording duration were classified as exhibiting sensor saturation and were removed.

Physiological noise from cardiac pulsation, respiration, and myogenic activity can contribute substantially to fNIRS signals, corresponding approximately to frequency bands of 0.6 to 1.6 Hz, 0.2 to 0.4 Hz, and 0.06 to 0.1 Hz, respectively (Reddy et al., 2021). To attenuate these sources of interference, a bandpass filter based on discrete cosine transform was applied within the range of 0.005 to 0.1 Hz, thereby removing low-frequency drift below 0.005 Hz and high-frequency noise above 0.1 Hz.

#### 2.4.2 Eye-tracking data

The eye-tracking system was used to record participants’ gaze patterns, including fixation duration and blink frequency, to assess ocular movement related to attentional engagement and potential indicators of boredom. Among various techniques for measuring eye movements, recent systems commonly employ the Pupil Center Corneal Reflection technique, in which an infrared light source illuminates the pupil and the corneal surface produces a detectable reflection. An infrared-sensitive camera captures these reflections, and the relative vector between the corneal reflection and the pupil center is computed to determine gaze direction (Punde et al., 2017). Thus, the eye-tracking device measures gaze position by detecting the reflection of emitted light on the surface of the eye.

A range of gaze-based metrics can be derived from eye-tracking data, including fixations, saccades, fixation sequences, and heat maps, each reflecting different characteristics of visual attention (Punde et al., 2017). In the present study, the Tobii Pro Spectrum system manufactured by Tobii Technology AB in Stockholm, Sweden was employed. This device uses infrared illumination to generate corneal reflection patterns and allows data acquisition at sampling frequencies of 150, 300, 600, or 1,200 Hz (Nyström et al., 2021). Data were collected at 1200 Hz to maximize temporal resolution.

Preprocessing was performed to ensure signal reliability and consistency. Periods in which gaze coordinates were not recorded for more than 100 ms were classified as blinks or off-screen gaze and were excluded from further analysis. Additionally, time points with a Tobii Pro Spectrum validity score of 2 or higher were considered unreliable due to unstable eye-position estimation and were treated as missing data (Nyström et al., 2021). These procedures minimized the impact of signal loss and noise-related errors on subsequent analyses. The resulting data were categorized into four analytic domains: temporal processing, fixation metrics, pupil response, and visit and glance metrics.

Visual stimuli were presented at a resolution of 1920 × 1,080 pixels. The AOI was defined as a centrally located region measuring 1,022 × 775 pixels, corresponding to approximately 38.2 percent of the display. This AOI specification ensured that fixation-based measures reflected attention directed toward the critical visual content rather than peripheral or unintended visual elements. The AOI boundaries were kept constant across all stimuli to maintain consistency and comparability across participants and conditions.

#### 2.4.3 Questionnaire data

Each set included three questionnaire question types. The first asked participants to indicate which version, chromatic or achromatic, they considered more appropriate in terms of color. The second required them to select which version appeared more esthetically attractive. The third asked which version elicited a stronger sense of immersion. Together, these question types were designed to assess the influence of color presence or absence on participants perception and appreciation of the two versions of the landscape paintings.

The Match and Feel were conceptually interrelated, reflecting the relationship between attentional focus and preference. The distinction between stimuli that participants reported liking or disliking emotionally was interpreted as an indicator of preference, which in turn influenced positive or negative evaluations. In the context of esthetic responses, artworks that were cognitively easier to process were more likely to be judged positively (Pelowski et al., 2016). To minimize potential bias in preference, well-known masterpieces were excluded from the stimulus set in the present study.

Therefore, in the present study, representative landscape paintings by well-known artists were excluded from the selection of experimental stimuli to minimize potential bias arising from prior familiarity. With respect to the third questionnaire question type on “immersion,” Hawkins and Edwards proposed that the act of “seeing” is intrinsically linked to the immersive experience, suggesting that concentrated observation of an image may induce a psychological state of unity between the viewer and the artwork (Kim, 2015).

### 2.5 Statistical analysis

All statistical analyses were performed using Stata/BE 19.5 (StataCorp LLC, College Station, TX, USA). Categorical variables such as gender were summarized using frequencies and percentages, while continuous variables such as age were reported as mean ± standard deviation (SD). To compare differences between Condition A and Condition B, independent-sample *t*-tests were conducted. Homogeneity of variance was assessed using the Levene test; if this assumption was violated, Welch’s *t*-test was applied instead. Given the multidimensional characteristics of the eye-tracking data, False Discovery Rate (FDR) correction was applied across the 14 key eye-tracking metrics listed in Table 1 to control for Type I error. Continuous outcomes were reported as mean ± SD, and between-condition differences were expressed in terms of mean difference, 95% confidence interval (CI), *t*-value, and *p*-raw.

**Table 1:** Eye-tracking metrics comparing Condition A and Condition B exposure sequences.

Category	Variable	Condition A Mean±SD	Condition B Mean±SD	Diff	95% CI	*t*-value	*p*-raw	p_FDR_
Temporal processing	Time to first fixation (MillIseconds)	156.2 ± 301.7	369.7 ± 1329.8	−213.6	[−366.4, −60.7]	−2.75	0.006**	0.0327*
	Time to first whole fixation (MillIseconds)	711.5 ± 797.9	958.2 ± 1374.5	−246.7	[−425.4, −68.1]	−2.71	0.007**	0.0327*
	Time to first Glance	153.1 ± 299.1	366.2 ± 1329.2	−213.0	[−365.7, −60.4]	−2.74	0.006**	0.0327*
	Duration of first fixation	288.0 ± 258.7	298.6 ± 211.2	−10.6	[−47.9, 26.7]	−0.56	0.577	0.5770
AOI Fixation metrics	Total duration of fixations (MillIseconds)	10273.9 ± 2923.7	9672.2 ± 3194.4	+601.7	[118.7, 1084.7]	2.45	0.015*	0.0451*
	Maximum duration of fixations (MillIseconds)	1033.1 ± 733.4	928.9 ± 468.9	+104.2	[7.0, 201.4]	2.11	0.036*	0.0504
	Total duration of whole fixation (MillIseconds)	7323.5 ± 3311.1	6728.9 ± 3265.9	+594.6	[75.9, 1113.3]	2.25	0.025*	0.0451*
	Average duration of fixations	309.8 ± 116.0	296.7 ± 100.1	+13.1	[−4.0, 30.2]	1.50	0.133	0.1690
AOI Pupil response	Average pupil diameter (MillIseconds)	3.51 ± 0.61	3.39 ± 0.60	+0.12	[0.02, 0.21]	2.40	0.017*	0.0451*
	Average whole fixation pupil dia (MillIseconds)	3.50 ± 0.61	3.39 ± 0.61	+0.11	[0.01, 0.20]	2.21	0.027*	0.0451*
	Average eye openness	8.61 ± 1.18	8.53 ± 1.14	+0.08	[−0.10, 0.27]	0.89	0.373	0.4020
AOI Visit/Glance metrics	Total duration of Visit (MillIseconds)	14308.7 ± 1544.0	13933.7 ± 2579.9	+375.0	[39.5, 710.5]	2.20	0.029*	0.0451*
	Total duration of Glances (MillIseconds)	14320.7 ± 1533.0	13948.8 ± 2574.5	+372.0	[37.6, 706.3]	2.19	0.029*	0.0451*
	Number of Visits	1.25 ± 0.68	1.33 ± 0.80	−0.07	[−0.19, 0.05]	−1.19	0.236	0.2570

Values are presented as Mean ± SD. Independent-sample *t*-test with unequal variances. * *p*-raw < 0.05, ** *p*-raw < 0.01, *pFDR < 0.05. The pFDR represents the *p*-raw adjusted for multiple comparisons using the False Discovery Rate correction. Condition A (achromatic to chromatic exposure) and Condition B (chromatic to achromatic exposure). AOI (Area of Interest) refers to predefined regions within the visual stimulus used to quantify fixation, pupil, and visit metrics. Temporal Processing refers to time-related visual processing measures such as time to first fixation. AOI Fixation Metrics refer to fixation measures within areas of interest such as fixation count and total duration. AOI Pupil Response represents pupil size measures within areas of interest such as mean diameter and dilation. AOI Visit/Glance Metrics indicate visit or glance measures within areas of interest such as number of visits and glance duration.

fNIRS data were processed in NIRSIT Quest using a General Linear Model framework. For each channel, changes in HbO and HbR concentrations were estimated. Paired-sample *t*-tests were used to evaluate differences between conditions for each channel, and *t*-values and *p*-raw were reported. To account for multiple comparisons across channels, FDR correction was applied to the full set of *p*-raw.

To examine the relationship between hemodynamic responses and gaze behavior, correlation and regression analyses were conducted between fNIRS-derived indices (HbO and HbR) and eye-tracking variables. Pairwise correlation coefficients were adjusted for multiple comparisons using FDR correction. Subsequently, robust regression models were employed to evaluate predictive relationships between the two modalities. For all multimodal analyses, FDR-adjusted *p*-raw were used to ensure statistical reliability and interpretive validity. All tests were two-tailed, and the significance threshold was set at *p* < 0.05 before and after correction.

## 3 Results

### 3.1 fNIRS

Paired-sample *t*-tests were conducted to compare differences in hemodynamic responses between Condition A and Condition B. Based on Brodmann area grouping, significant increases in HbO were observed in the left DLPF (*p*-raw = 0.006), the left OFC (*p*-raw = 0.043), and the right OFC (*p*-raw = 0.043). All of these *p*-raw were below the significance threshold of 0.05, indicating greater HbO activation in Condition A compared to Condition B. Moreover, all significant channels exhibited positive *t*-values, demonstrating a consistent direction of increased activation. Notably, the left DLPFC effect remained significant following False Discovery Rate correction (*p*-raw = 0.006, pFDR = 0.050), indicating that the observed difference reflects a reliable and non-random effect at both the individual test and multiple-comparison levels.

In contrast, no significant differences were observed for HbR, as all *p*-raw exceeded 0.05. The corresponding pFDR values for HbR also exceeded the 0.05 threshold, indicating the absence of statistically reliable condition-related effects in HbR responses. According to established criteria, only results with pFDR ≤ 0.05 were considered statistically significant (Reddy et al., 2021). The significant HbO effects across the left DLPFC, left OFC, and right OFC together indicate that the condition-related activation differences were distributed across both hemispheres rather than localized to a single cortical region. These results are summarized in Table 2.

**Table 2:** fNIRS HbO and HbR responses comparing Condition A and Condition B exposure sequences.

Region	HbO					HbR				
	Condition A Mean±SD	Condition B Mean±SD	95% CI	*p*-raw	p_FDR_	Condition A Mean±SD	Condition B Mean±SD	95% CI	*p*-raw	p_FDR_
Left DLPFC	0.006 ± 0.044	−0.005 ± 0.046	[−0.014, 0.015]	0.006 **	0.050*	0.002 ± 0.019	0.007 ± 0.029	[−0.003, 0.012]	0.257	0.677
Right DLPFC	0.009 ± 0.053	−0.014 ± 0.047	[−0.019, 0.014]	0.118	0.189	−0.010 ± 0.037	0.010 ± 0.018	[−0.010, 0.010]	0.977	0.977
Left FPC	0.011 ± 0.047	−0.008 ± 0.040	[−0.013, 0.016]	0.058	0.116	−0.006 ± 0.015	0.008 ± 0.015	[−0.004, 0.006]	0.338	0.677
Right FPC	0.004 ± 0.045	−0.006 ± 0.045	[−0.016, 0.014]	0.459	0.612	0.003 ± 0.013	0.007 ± 0.012	[0.001, 0.009]	0.055	0.441
Left VLPFC	−0.011 ± 0.040	−0.016 ± 0.045	[−0.025, −0.001]	0.600	0.686	0.012 ± 0.032	0.011 ± 0.019	[0.003, 0.020]	0.776	0.962
Right VLPFC	0.001 ± 0.031	−0.008 ± 0.037	[−0.017, 0.009]	0.749	0.749	0.001 ± 0.036	0.006 ± 0.018	[−0.001, 0.018]	0.302	0.677
Left OFC	0.005 ± 0.042	−0.009 ± 0.039	[−0.015, 0.011]	0.043*	0.115	−0.001 ± 0.014	0.008 ± 0.011	[−0.003, 0.005]	0.842	0.962
Right OFC	0.005 ± 0.042	−0.009 ± 0.039	[−0.015, 0.011]	0.043*	0.115	−0.001 ± 0.014	0.008 ± 0.011	[−0.001, 0.008]	0.842	0.962

Values are presented as Mean ± SD. Statistical significance is indicated as follows: *p-raw < 0.05, **p-raw < 0.01. The 95% confidence intervals (CI) represent estimated mean differences between conditions. pFDR values were adjusted for multiple comparisons using the false discovery rate (FDR) correction. Condition A (achromatic to chromatic exposure) and Condition B (chromatic to achromatic exposure). HbO (oxyhemoglobin) and HbR (deoxyhemoglobin). fNIRS measurement sites: (1) Right DLPFC (Right dorsolateral prefrontal cortex); (2) Left DLPFC (Left dorsolateral prefrontal cortex); (3) Right FPC (Right frontopolar cortex); (4) Left FPC (Left frontopolar cortex); (5) Right VLPFC (Right ventrolateral prefrontal cortex); (6) Left VLPFC (Left ventrolateral prefrontal cortex).

Within the left DLPFC, significant differences were observed in channels 33 (*p*-raw = 0.033), 34 (*p*-raw = 0.039), 35 (*p*-raw = 0.047), 38 (*p*-raw = 0.038), and 43 (*p*-raw = 0.030). In addition, significant activation differences were identified across multiple channels in the left OFC (channels 14, 15, 16, 29, and 30) and the right OFC (channels 31, 32, 46, 47, and 48), each demonstrating *p* = 0.043. Further significant effects were also observed in channel 3 (*p*-raw = 0.047) and channel 23 (*p*-raw = 0.025). However, none of these channels retained significance following False Discovery Rate adjustment, indicating that these effects did not survive correction for multiple comparisons.

**Figure 4:**
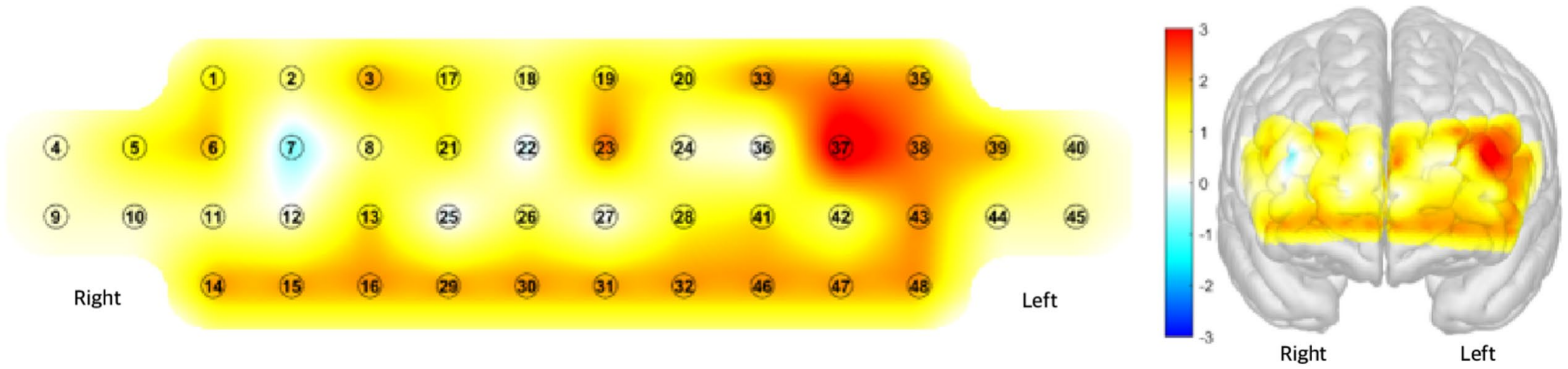
Paried sample *t*-test student’s t of HbO (Condition A − Condition B). Warmer colors, ranging from yellow to red, indicate higher positive *t*-values, reflecting greater changes in oxyhemoglobin (HbO) concentration during Condition A compared to Condition B. In contrast, cooler colors such as blue indicate negative *t*-values, representing reduced HbO changes during Condition A. Significant positive activations were primarily observed in the warmer regions of the map, corresponding to the Right DLPFC (Right dorsolateral prefrontal cortex), Left OFC (Left orbitofrontal cortex), and Right OFC (Right orbitofrontal cortex).

All significant channels exhibited positive *t*-values, indicating consistently greater activation in Condition A relative to Condition B. This pattern suggests that Condition A elicited stronger hemodynamic responses across multiple subregions of the prefrontal cortex. In the visualization, these increases are represented by warmer color gradients, indicating elevated HbO concentration in Figure 4.

**Figure 5:**
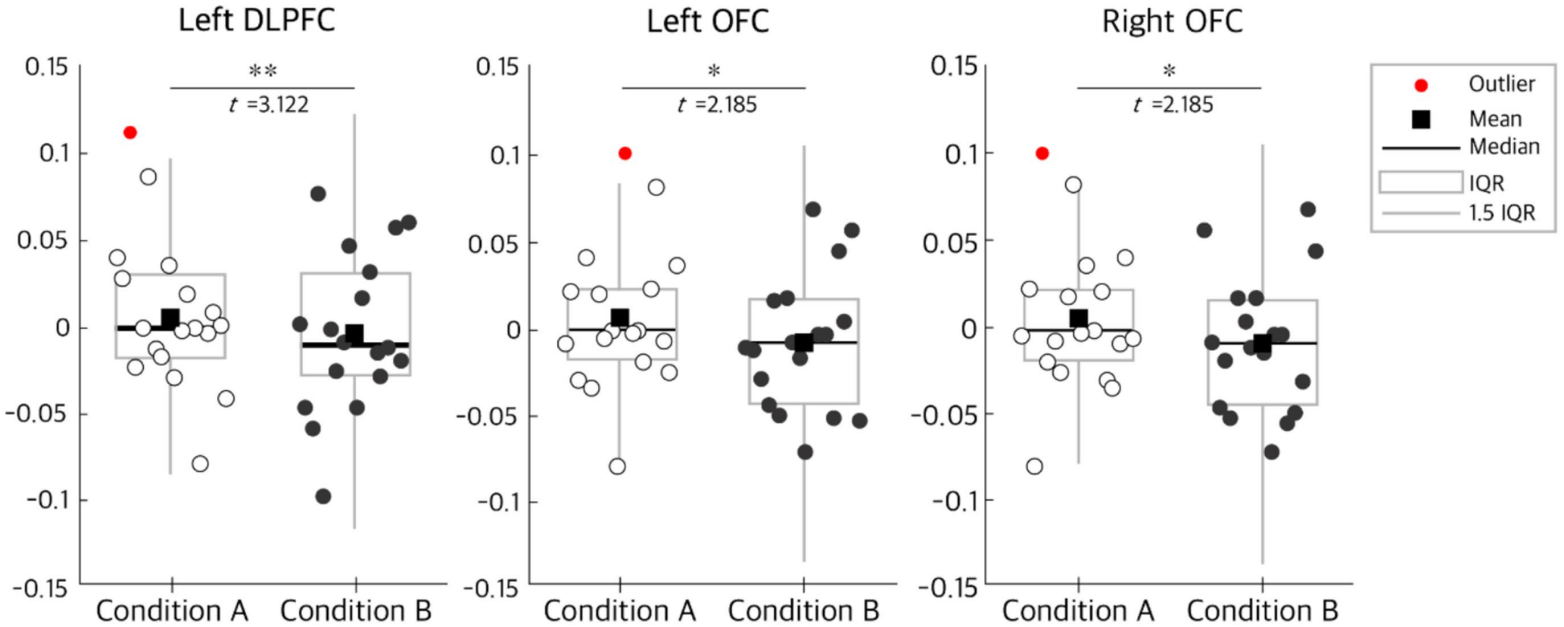
Distribution of HbO activation in significant prefrontal cortex regions by color exposure order. Significant prefrontal cortex regions are defined as follows: Left DLPFC (Left dorsolateral prefrontal cortex), Left OFC (Left orbitofrontal cortex), and Right OFC (Right orbitofrontal cortex). Condition A represents achromatic to chromatic exposure, and Condition B represents chromatic to achromatic exposure. Statistical analysis was performed using paired-samples *t*-tests, with significance indicated as *p-raw < 0.05, **p-raw < 0.01. The *t*-values reported below correspond to the paired-samples *t*-test results: Left DLPFC, *t* = 3.122; Left OFC, *t* = 2.185; and Right OFC, *t* = 2.185. In the boxplots, the central line represents the median, the square denotes the mean, whiskers indicate 1.5 × IQR, and crosses mark outliers. The distribution converges toward the median, with most data points clustered within the interquartile range (IQR).

The boxplot results further illustrate the distributional patterns of activation. Both the mean and median values under Condition A were higher than those under Condition B across the Left DLPFC, Left OFC, and Right OFC. When examining the ranges defined by 1.5 IQR, the overall distributions for Condition A were consistently shifted toward higher values relative to Condition B. Notably, the elevated central tendency observed in the Left DLPFC and Left OFC was mirrored in the Right OFC, reinforcing the pattern of stronger activation under Condition A across all examined regions in Figure 5.

### 3.2 Eye-tracker

The results were organized into four domains. In the Temporal Processing domain, all relevant measures showed pFDR < 0.05, indicating that Condition B elicited faster initial visual responses than Condition A. In contrast, the AOI Fixation Metrics domain showed longer fixation durations in Condition A (pFDR < 0.05), with Total duration of whole fixation remaining significant after correction, suggesting more sustained visual attention under Condition A.

In the AOI Pupil Response domain, both pupil metrics were significantly higher in Condition A (pFDR < 0.05), implying greater arousal and/or cognitive processing demands during chromatic stimulus viewing. Finally, in the AOI Visit/Glance Metrics domain, Total duration of all visits and glances were also greater in Condition A (pFDR < 0.05), indicating more persistent visual exploration. Overall, Condition A was associated with sustained attentional engagement, whereas Condition B was associated with faster initial orienting responses in Table 1.

Previous research has shown that individuals demonstrate longer fixation durations, more frequent fixations, and larger pupil diameters when viewing preferred colors or emotionally salient stimuli (Lee et al., 2005). The present findings are consistent with this pattern: participants who viewed chromatic stimuli first exhibited greater fixation persistence and greater pupil dilation than when viewing achromatic stimuli. This suggests that chromatic presentation elicited stronger visual attention and emotional arousal, resulting in more prolonged and pronounced gaze responses compared to achromatic presentation.

**Figure 6:**
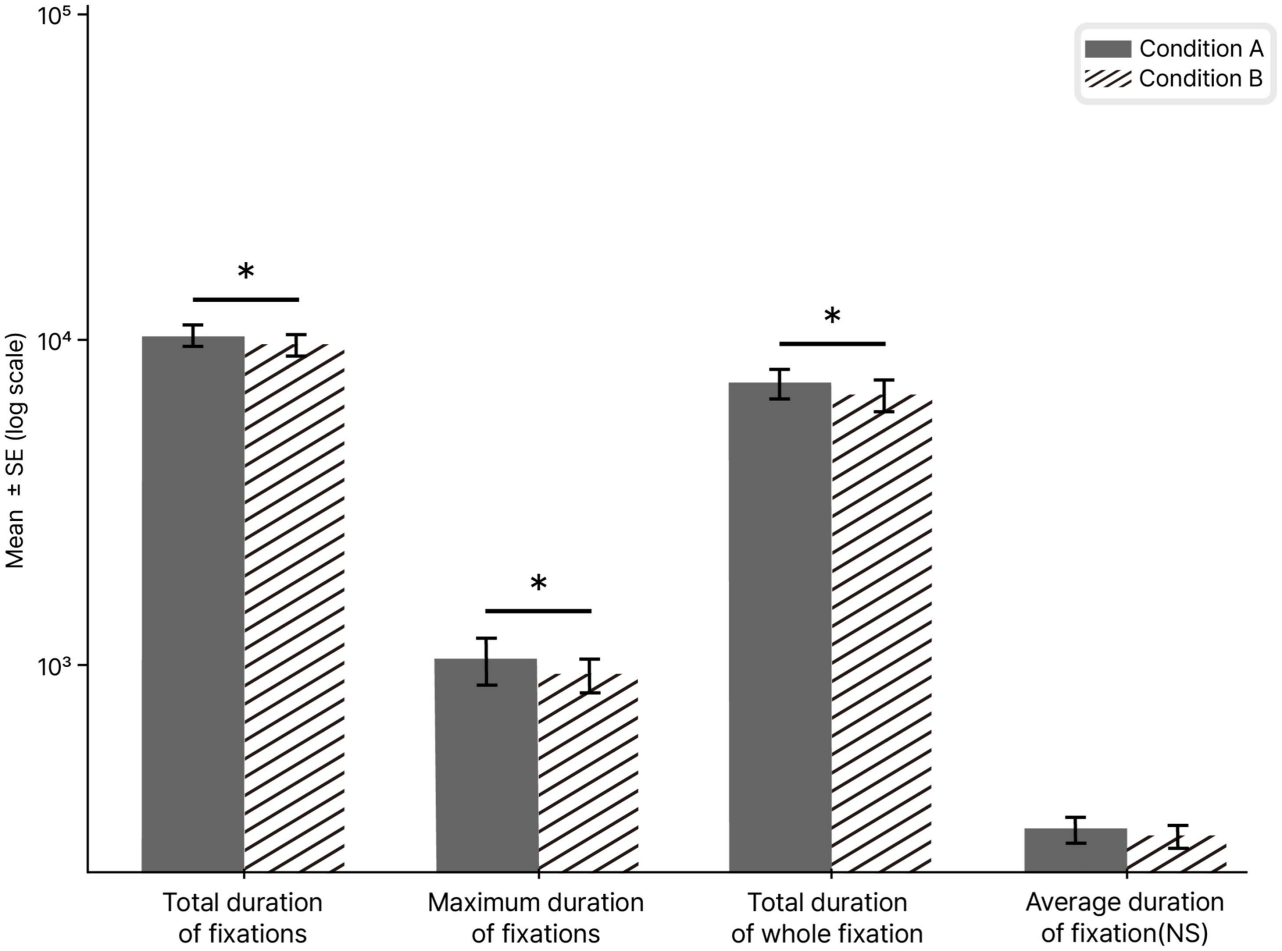
Comparison of eye-tracking fixation metrics between Condition A and Condition B. Condition A (achromatic to chromatic exposure) and Condition B (chromatic to achromatic exposure). Total duration of fixations refers to the sum of all fixation durations, maximum duration of fixations indicates the longest single fixation, total duration of whole fixation represents the cumulative duration of consecutive fixations within an AOI, and average duration of fixations denotes the mean fixation duration, which was not statistically significant. Independent-sample *t*-test with unequal variances: statistical significance was defined as *p-raw < 0.05 and **p-raw < 0.01.

Significant results within the AOI Fixation Metrics category were identified for Total duration of fixations (*p*-raw = 0.015), Maximum duration of fixations (*p*-raw = 0.036), and Total duration of whole fixation (*p*-raw = 0.025) in Figure 6. Consistent with prior research, visual regions of interest are typically characterized by key indicators derived from eye-movement records, such as fixations, scanpaths, and focal attention areas (Santella and DeCarlo, 2004).

### 3.3 Questionnaire

**Figure 7:**
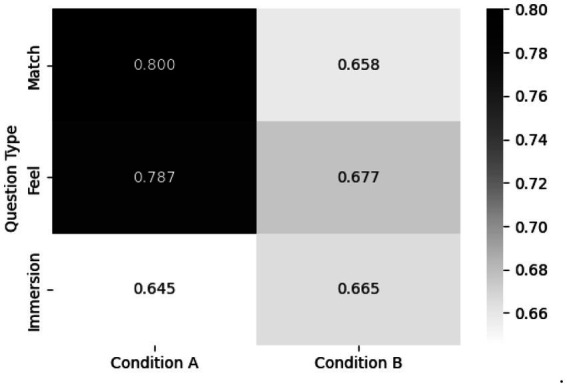
Comparison of questionnaire responses across color exposure conditions. The figure show questionnaire results comparing Condition A and Condition B across three categories: Match, Feel and Immersion. The x-axis indicates the experimental conditions, and the y-axis denotes the question types. Numeric values within each cell represent the mean scores, and shading intensity reflects their magnitude, with darker shading corresponding to higher scores.

The questionnaire results demonstrated that, overall, Condition A yielded higher response scores than Condition B. For the Match question type, Condition A achieved a mean score of 0.800 compared with 0.658 for Condition B, representing the largest difference observed between the two conditions. Similarly, for the Feel question type, Condition A scored 0.787, whereas Condition B scored 0.677, indicating greater emotional congruence under Condition A. By contrast, for the Immersion question type, Condition A recorded a score of 0.645 and Condition B a score of 0.665, showing only a negligible difference between the two conditions. For the Match and Feel question types, the mean values were consistently higher in Condition A than in Condition B, confirming greater agreement in Condition A. Moreover, both question types exhibited relatively small SE, suggesting high reliability of the estimates. Finally, the presence of positive *t*-values together with *p*-raw below.05 indicated that the differences between the two conditions were statistically significant in Figure 7 and Table 3.

**Table 3:** Comparison of questionnaire response scores between Condition A and Condition B.

Question type	Condition A Mean (SE)	Condition B Mean (SE)	Diff	95% CI	*t*-value	*p*-raw
Match	0.800 (0.032)	0.658 (0.038)	+0.142	[0.044, 0.240]	2.81	0.005**
Feel	0.787 (0.033)	0.677 (0.038)	+0.110	[0.012, 0.270]	2.18	0.029*
Immersion	0.645 (0.038)	0.665 (0.038)	−0.019	[−0.125, 0.086]	−0.36	0.720

Values are presented as proportion (standard error); Cl = 95% confidence interval for the difference between groups; *p*-raw from two-sample *z*-test for proportions; * *p*-raw < 0.05, ** *p*-raw < 0.01. Condition A (achromatic to chromatic exposure) and Condition B (chromatic to achromatic exposure). Match refers to perceived alignment between stimulus and context, Feel indicates the affective response elicited by the stimulus, and Immersion represents the extent of cognitive and emotional absorption in the stimulus.

### 3.4 Multimodal analysis

To examine the relationships among neural activation, gaze behavior, and subjective experience, within-subject correlation analyses were performed using condition difference scores (*Δ* = A − B). A significant positive correlation was observed between changes in orbitofrontal cortex activation and subjective esthetic feeling ratings. Specifically, both the left OFC (*p*-raw = 0.008) and right OFC (*p*-raw = 0.008) demonstrated very strong correlations with the Feel rating (*p*-raw = 0.990), indicating statistical significance at *p*-raw < 0.01. These findings suggest that esthetic emotional experience is closely linked to the evaluative and reward-related functions of the orbitofrontal cortex. Moreover, the presence of similar effects in both hemispheres indicates that the affective appraisal of visual artworks engages a bilateral prefrontal network rather than a lateralized response. In contrast, correlations involving the left DLPFC (*p*-raw = 0.217), left OFC (*p*-raw = 0.348), and right OFC (*p*-raw = 0.348) with measures of cognitive immersion and perceived congruence were not statistically significant, suggesting that these cognitive components may have weaker neural coupling compared to affective valuation.

Among the eye-tracking measures, average pupil diameter showed a significant positive correlation with subjective feeling ratings (*p*-raw = 0.018), indicating that emotional engagement co-occurs with increased physiological arousal. Although Time to first fixation did not reach statistical significance (*p*-raw = 0.052), the marginal trend suggests a potential relationship between early attentional orienting and esthetic cognitive processing. Regression analysis further demonstrated that changes in left OFC activation were a strong predictor of subjective esthetic feeling, explaining 98.41 percent of the variance (R^2^ = 0.9841, *β* = 5.312). This finding highlights the close correspondence between neural valuation processes and self-reported emotional experience. Collectively, these multimodal results indicate that gaze-derived physiological markers and prefrontal hemodynamic activity can serve as objective indicators of esthetic affect and attention in Table 4.

**Table 4:** Correlations among fNIRS activations, eye-tracker measures, and questionnaire responses in the multimodal analysis.

Relation	Region	Question type	Pearson’s *r*	*p*-raw	p_FDR_
Core neuro-affective	Left OFC (HbO)	Feel	0.99	0.008**	0.096
Bilateral check	Right OFC (HbO)	Feel	0.99	0.008**	0.096
Exploratory link	Left DLPFC (HbO)	Match	0.78	0.217	0.71
	Left OFC (HbO)	Immersion	0.65	0.348	0.748
	Right OFC (HbO)	Immersion	0.65	0.348	0.748
Biometric-subjective	Average pupil diameter (MillIseconds)	Immersion	0.98	0.018*	0.331
	Time to first fixation (MillIseconds)	Match	0.95	0.052	0.748

False Discovery Rate (FDR) correction was applied to the *p*-raw, with m = 36. Coefficients of determination R^2^ and regression coefficients β are reported only for the strongest association identified through simple linear regression. Statistical significance: **p-raw < 0.01, *p-raw < 0.05.

## 4 Discussion

In this study, a combined fNIRS and eye-tracking approach was employed to quantify cognitive responses such as attention and immersion elicited during the viewing of chromatic and achromatic artworks, and to relate these objective indices to subjective ratings. The HbO results revealed significantly greater activation in the left DLPFC, left OFC, and right OFC in Condition A compared to Condition B, indicating that presenting chromatic stimuli after achromatic stimuli elicited stronger engagement of prefrontal functions associated with attentional allocation and cognitive integration. In particular, channels 33, 34, 35, 38, and 43 within the left DLPFC showed pronounced increases in HbO concentration. The dorsolateral prefrontal cortex has been implicated in emotion regulation and the modulation of affective response intensity, with reduced activation often associated with suppression of negative affect (Jahani et al., 2017; Han et al., 2024). Therefore, the enhanced left DLPFC activation observed here suggests that cognitive–emotional evaluative processes were more actively engaged in Condition A.

Bilateral activation in the OFC was also observed, with all channels in both the left and right OFC demonstrating increased hemodynamic response. Consistent with previous research, OFC activation is associated with cognitive control, affective evaluation, and adaptive modulation of emotional experience (Gu et al., 2021). Thus, the present findings indicate that chromatic-first presentation induced stronger affective appraisal and valuation processes than achromatic-first presentation. This effect extends beyond low-level perceptual processing, suggesting that the ordering of color exposure influences higher-order integrative functions related to meaning construction, memory retrieval, and evaluative judgment.

Furthermore, prior work has shown that the recognition of colored stimuli engages broader prefrontal networks and facilitates semantic association and interpretative reasoning (Bramão et al., 2010). In line with these findings, the current results demonstrate that introducing color at a later stage in the viewing sequence enhances prefrontal activation linked to cognitive integration and esthetic evaluation. Taken together, these findings suggest that the temporal ordering of chromatic information modulates both attentional and affective components of esthetic experience, engaging prefrontal networks that jointly support cognitive appraisal and emotional response.

The cognitive processing of visual stimuli is closely characterized by interactions between gaze behavior and prefrontal cortical activation. When visual features such as color contrast or stimulus order vary, large-scale fronto-parietal–temporal networks are engaged, with the right superior parietal lobule and the left medial prefrontal cortex serving as key hubs for attentional shifting and spatial exploratory control. The responsiveness of these systems plays a central role in esthetic perception, such that even identical stimuli may evoke stronger activation depending on individual differences in esthetic preference and immersive engagement. This pattern suggests that neural responses to visual art extend beyond sensory-level processing and reflect higher-order integrative and predictive cognitive mechanisms (Arioli et al., 2025).

**Figure 8:**
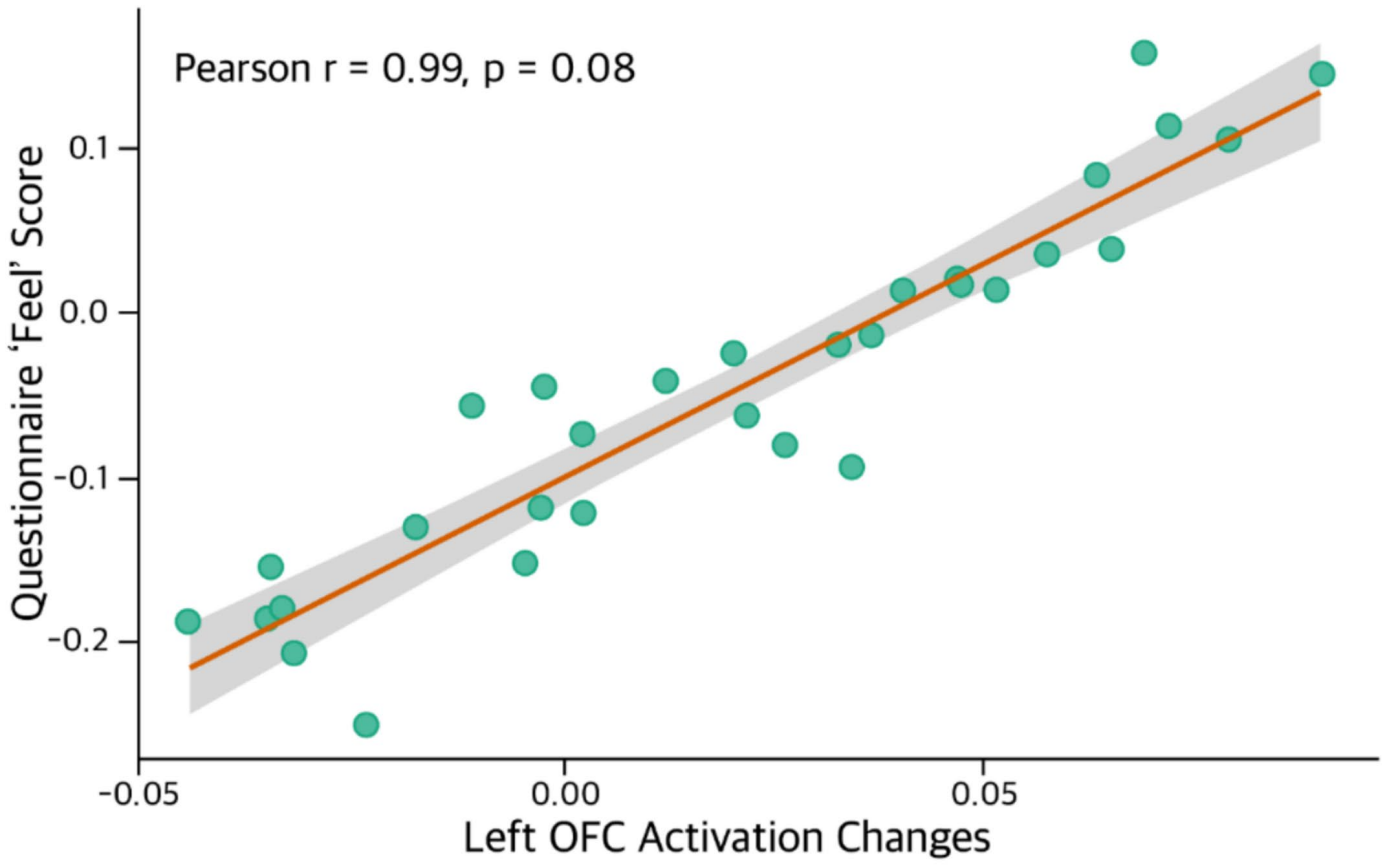
Correlation between Left OFC activation changes and subjective feel. The scatter plot illustrates the relationship between changes in left orbitofrontal cortex (OFC) activation and participants’ subjective ‘Feel’ scores derived from the post-stimulus questionnaire (*N* = 31). A strong positive correlation was observed between OFC activation and reported esthetic feeling (Pearson’s r = 0.99, *p*-raw = 0.08), indicating that greater OFC activation was associated with higher levels of positive esthetic experience. The shaded region represents the 95% confidence interval of the fitted regression line.

The integrated analysis of fNIRS, eye-tracking indices, and subjective self-report measures further demonstrated that visual attention, physiological arousal, and emotional appraisal operate in a tightly interdependent manner during esthetic experience. In particular, neural activation in the left orbitofrontal cortex exhibited a highly robust correlation with the esthetic Feel rating, and regression analysis indicated that changes in left OFC activation accounted for 98.41 percent of the variance in subjective emotional experience (R^2^ = 0.9841, *β* = 5.312). This finding highlights the OFC as a core neural substrate not only for perceptual evaluation but also for affective valuation and the experience of esthetic pleasure. Although this effect did not remain statistically significant following FDR correction, the magnitude of the observed association strongly suggests that the relationship is unlikely to be incidental in Figure 8.

The eye-tracking results indicated that the presentation order from Condition A significantly influenced both early attentional orientation and sustained visual processing. Reduced Time to First Fixation measures in Condition A suggest that gaze was initiated more rapidly when the chromatic stimulus followed the achromatic one, likely due to heightened contrast and novelty effects enhancing exploratory attention. Furthermore, increases in total fixation duration and maximum fixation duration indicate deeper sustained attention rather than merely greater frequency of gaze shifts, reflecting qualitative intensification of visual information processing (Francuz et al., 2018; Massaro et al., 2012; Fontoura and Menu, 2021). This pattern was consistent with the self-report findings: Condition A elicited significantly higher ratings on the Match and Feel items, suggesting stronger positive affective appraisal and attentional engagement. However, Immersion ratings did not differ substantially between conditions, indicating that color presentation order may exert a greater influence on early evaluative and emotional responses than on sustained narrative or experiential absorption. These results support the view that perceptual contrast and temporal sequencing act as key modulators of esthetic attention and affective appraisal, while prior knowledge and personal experience shape evaluative preference formation (Dupuy et al., 2024).

These affective and cognitive outcomes align closely with the neurophysiological findings. Recent work has demonstrated that fixation patterns correlate with functional brain responses in regions supporting attention and evaluative processing (Arioli et al., 2025), suggesting that visual attention is not a simple sensory response but is integrally linked to higher-order neural mechanisms. Within this framework, prior exposure to achromatic stimuli may establish a perceptual baseline against which subsequent chromatic stimuli are selectively prioritized. Sequential contextual effects have been shown to enhance sensitivity to visual contrast, lower the threshold for attentional selection, and facilitate the detection of novel visual features (Becker, 2010; Chun and Jiang, 1998). Thus, when chromatic stimuli are presented following achromatic ones, their relative salience increases, elevating autonomic arousal and activating attentional control circuits. This bottom-up attentional enhancement manifests physiologically through increased pupil diameter and longer fixation durations (Scholkmann et al., 2017). Therefore, the observed differences by color sequence reflect not merely sensory contrast, but underlying neurocognitive modulation of attention and evaluative processing.

In line with this interpretation, integrating neural activation indices with gaze behavior enables a more objective inference of subjective experiences during art viewing, reflecting a tight coupling between prefrontal mechanisms supporting cognitive–emotional integration and visual exploration patterns. To clarify the functional significance of these multimodal outcomes, we interpreted the results within four conceptual domains: the Core Neuro-Affective domain, which highlights the association between orbitofrontal cortex activation and esthetic valuation; the Bilateral Check domain, which examines hemispheric symmetry across the orbitofrontal cortex to determine whether affective evaluations are lateralized or coordinated; the Exploratory Link domain, which encompasses attentional orientation, perceived congruence, and immersive engagement to illustrate how prefrontal activation contributes to integrative evaluative processes beyond affective appraisal alone; and the Biometric–Subjective domain, which connects physiological indicators derived from eye-tracking, such as pupil dilation and fixation duration, with subjective experiential states, demonstrating how nonverbal biometric signals align with internally perceived esthetic engagement in Table 4.

This study has several limitations. First, the simultaneous use of fNIRS and eye-tracking equipment may have caused physical discomfort, due to device weight, sensor placement, and restricted head or body movement, potentially affecting natural viewing behavior. Additionally, sustaining visual fixation on a monitor for more than 15 min may have introduced visual fatigue for some participants. Second, the relatively small sample size may limit generalizability of the findings. Third, the experiment focused on a single genre of visual art, which may not represent the diversity of esthetic contexts. Fourth, the participant sample consisted primarily of young adults, limiting applicability to broader age groups. Nonetheless, the study holds practical relevance for applications in museum and gallery display strategies, commercial space visual design, and user-centered cultural experience research.

## 5 Conclusion

This study demonstrated that viewing chromatic stimuli after achromatic stimuli elicited significantly greater activation in the left DLPFC, left OFC, and right OFC, as measured by fNIRS, alongside consistent differences in eye-tracking indicators such as fixation duration, fixation frequency, and gaze engagement, as well as subjective esthetic ratings. These findings suggest that chromatic artworks evoke stronger interest and attentional involvement compared to achromatic artworks during esthetic appreciation. Moreover, the correlation analysis across fNIRS, eye-tracking, and self-report measures revealed that changes in OFC activation were closely associated with affective esthetic responses, indicating that color influences not only cognitive processing but also the intensity of emotional experience. Collectively, these results support the role of color as a key integrative factor in modulating emotional engagement and esthetic judgment. Future research may further explore the neurocognitive mechanisms underlying color-based modulation to inform museum exhibition strategies and the design of esthetic experiences in real-world cultural environments.

## Data Availability

The raw data supporting the findings of this study are available from the corresponding author upon reasonable request.
